# eVIDENCE: a practical variant filtering for low-frequency variants detection in cell-free DNA

**DOI:** 10.1038/s41598-019-51459-4

**Published:** 2019-10-22

**Authors:** Kei Mizuno, Shusuke Akamatsu, Takayuki Sumiyoshi, Jing Hao Wong, Masashi Fujita, Kazuaki Maejima, Kaoru Nakano, Atushi Ono, Hiroshi Aikata, Masaki Ueno, Shinya Hayami, Hiroki Yamaue, Kazuaki Chayama, Takahiro Inoue, Osamu Ogawa, Hidewaki Nakagawa, Akihiro Fujimoto

**Affiliations:** 1 Department of Urology, Kyoto University Graduate School of Medicine, Kyoto, Japan; 2 Department of Drug Discovery Medicine, Kyoto University Graduate School of Medicine, Kyoto, Japan; 3 Laboratory for Cancer Genomics, RIKEN Center for Integrative Medical Sciences, Yokohama, Japan; 4 Department of Gastroenterology and Metabolism, Graduate School of Biomedical and Health Sciences, Hiroshima University, Hiroshima, Japan; 5 Second Department of Surgery, Wakayama Medical University, Wakayama, Japan; 6 Department of Human Genetics, The University of Tokyo, Graduate School of Medicine, Tokyo, Japan

**Keywords:** Tumour biomarkers, Genome informatics, Cancer genetics

## Abstract

Plasma cell-free DNA (cfDNA) testing plays an increasingly important role in precision medicine for cancer. However, circulating cell-free tumor DNA (ctDNA) is highly diluted by cfDNA from non-cancer cells, complicating ctDNA detection and analysis. To identify low-frequency variants, we developed a program, eVIDENCE, which is a workflow for filtering candidate variants detected by using the ThruPLEX tag-seq (Takara Bio), a commercially-available molecular barcoding kit. We analyzed 27 cfDNA samples from hepatocellular carcinoma patients. Sequencing libraries were constructed and hybridized to our custom panel targeting about 80 genes. An initial variant calling identified 36,500 single nucleotide variants (SNVs) and 9,300 insertions and deletions (indels) across the 27 samples, but the number was much greater than expected when compared with previous cancer genome studies. eVIDENCE was applied to the candidate variants and finally 70 SNVs and 7 indels remained. Of the 77 variants, 49 (63.6%) showed VAF of < 1% (0.20–0.98%). Twenty-five variants were selected in an unbiased manner and all were successfully validated, suggesting that eVIDENCE can identify variants with VAF of ≥ 0.2%. Additionally, this study is the first to detect hepatitis B virus integration sites and genomic rearrangements in the *TERT* region from cfDNA of HCC patients. We consider that our method can be applied in the examination of cfDNA from other types of malignancies using specific custom gene panels and will contribute to comprehensive ctDNA analysis.

## Introduction

Precision medicine in cancer treatment is an approach to select the most accurate and effective therapeutic agents to treat each patient’s cancer based on a genetic understanding of the tumor, as well as the individual. Next-generation sequencing (NGS) enables multiplex genomic testing from a single tissue sample, which assists clinicians in choosing the most appropriate targeted treatment. In hepatocellular carcinoma (HCC), however, diagnosis is often done in the absence of tumor biopsy, and on the basis of imaging studies such as multiphasic helical computed tomography or magnetic resonance imaging. Therefore, in patients with advanced HCC, prevalent adoption of tissue-based NGS testing for precision medicine remains challenging.

Recently, analysis of circulating cell-free tumor DNA (ctDNA) is gaining significant attention as a minimally-invasive tool for biomarker discovery. CtDNA is released into the blood by apoptosis and necrosis of cancer cells, and is a constituent of circulating cell-free DNA (cfDNA)^1^. CfDNA testing is theoretically available to any patient and can be sequentially performed to observe the current genetic profile of tumor that may change during treatment^2^. Furthermore, ctDNA could capture inter- and intra-tumor heterogeneity, unlike tissue biopsy from only one region^3,4^. Therefore, ctDNA analysis could be an effective tool for molecular profiling in unresectable HCC with multiple intrahepatic lesions. However, ctDNA is highly diluted by cfDNA from non-cancer cells and can be present at allele fractions below 0.5%, complicating ctDNA detection and analysis^5^. Although NGS of ctDNA can reveal comprehensive genomic alterations, it is a key challenge to distinguish variants at such low fraction from background errors of sequencing.

In recent years, several reports have showed improved detection limits of ctDNA using targeted sequencing approaches^6–17^. In 2012, Forshew *et al*.^6^ reported TAm-seq, which is a polymerase chain reaction (PCR) amplicon deep sequencing targeting 6 genes, and the analytical sensitivity of this technology was shown to be down to 0.14% variant allele frequency (VAF). Gale *et al*.^7^ developed an enhanced version of TAm-seq technology (eTAm-seq), targeting hotspots of 31 genes and entire coding regions of 4 genes. This assay achieved a detection limit of 0.02% in cfDNA. Furthermore in 2014, Newman *et al*.^8^ described a method for quantifying ctDNA by capture enrichment sequencing, called CAPP-seq. With information about recurrently mutated regions in the cancer of interest and tumor genotypes from sequencing of tumor biopsies, this method identified variants with VAF of 0.02%. Moreover, the technology was improved by molecular barcoding and characterizing the stereotyped background artifacts for error suppression and showed a detection limit down to 0.0025%, tenfold below the original method^9^. Molecular barcoding was developed^18^ to identify original fragments by de-duplicating the sequencing reads that might contain PCR amplification and/or sequencing errors, and has been applied to several ctDNA analysis methods^9–12^.

Although these reported methods identifying low-frequency variants represented high analytical performance, the main limitation is that most of them interrogated hotspots or limited loci, which might result in missing variants in genes that lack hotspots such as tumor suppressors. On the other hand, Lanman *et al*.^10^ developed a highly sensitive and highly specific platform for comprehensive ctDNA analysis, called Guardant360. Guardant360, at the time of this study, targets complete exons of 73 cancer-related genes. It was recently reported that the 95% limit of detection of the platform for single nucleotide variants (SNVs) and insertions and deletions (indels) was 0.25% and 0.2% VAF, respectively, and its reportable range for SNVs and indels was ≥0.04% and ≥0.02% VAF, respectively^19^. However, a recent study comparing Guardant360 with PlasmaSELECT, another highly accurate, specific and sensitive platform reported that concordance of reported gene alterations in the same patients with prostate cancer was very low^20^, suggesting that the analysis method for detecting rare variants from comprehensive genomic testing of cfDNA has yet to be well-established. We therefore sought to develop a ctDNA analysis method using molecular barcodes and optimized bioinformatics methods to detect low-frequency variants from sequencing data targeting about 80 genes.

In the current study, we introduce eVIDENCE (enhanced Variant IDENtifier for CEll-free DNA), an approach to reduce false positive calls and identify low-frequency variants from cfDNA sequencing data with high specificity and sensitivity, using the ThruPLEX tag-seq (Takara Bio), a commercially-available molecular barcoding kit. To examine our algorithm, we performed sequencing of an artificial library generated by mixing three libraries with different fractions. Then, we applied this method for the analysis of targeted sequencing data of cfDNA from HCC patients and identified variants. A portion of the detected variants were selected in an unbiased manner and subjected to validation experiments to assess the method’s specificity. We also compared the identified variants with the sequencing results of the matched tumor DNA samples to validate its sensitivity. Additionally, we analyzed known important structural variations (hepatitis B virus (HBV) integration sites and genomic rearrangements in the *TERT* region). This study shows that our method can be clinically utilized for ctDNA analysis in the HCC field. The source code of eVIDENCE is freely available at https://github.com/mizunokei/eVIDENCE.

## Results

### Targeted capture sequencing

We sequenced cfDNA from HCC patients and artificial library (see below). The input DNA fragments were uniquely tagged and NGS libraries with Illumina adapters were constructed using ThruPLEX Tag-seq (Takara Bio) (Supplementary Fig. S1). We analyzed targeted exonic regions and splice sites of 79 genes and the *TERT* promoter region (Supplementary Table S1). Sequencing reads were mapped to the human reference genome, and the BAM files were processed using Connor (https://github.com/umich-brcf-bioinf/Connor), an open source software for combining sequences where the alignment structure and molecular barcodes match resulting in a new BAM file with consensus sequences.

### Development of eVIDENCE method and its evaluation

**Figure 1:**
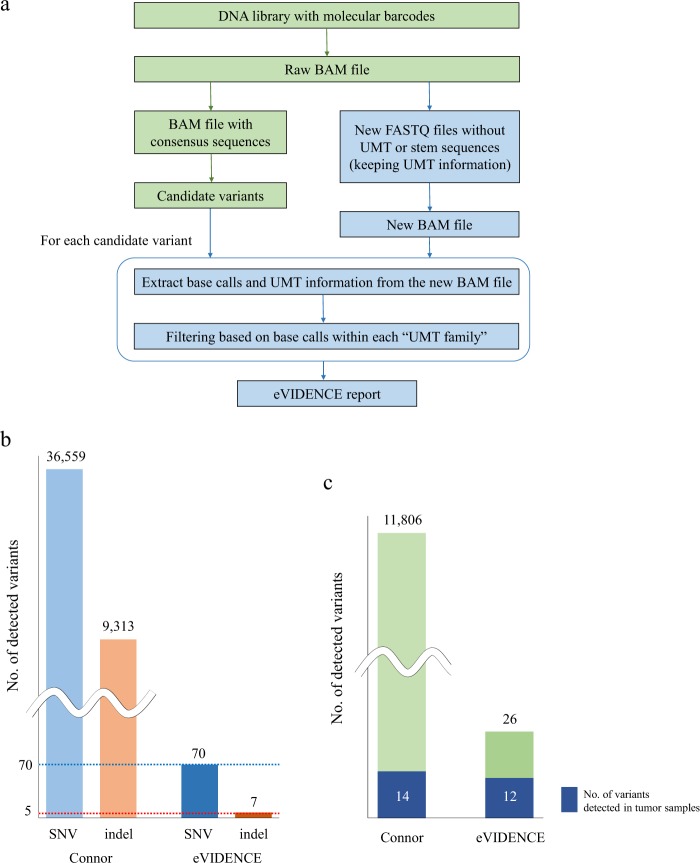
(**a**) Summary of the eVIDENCE pipeline. An input BAM file is converted to the BAM file with consensus alignment pairs using Connor. Candidate variants are called using the converted BAM file. eVIDENCE removes unique molecular tag (UMT) and stem sequences from a raw BAM file and creates new FASTQ files while retaining the UMT information. These FASTQ files are converted into a new BAM file and for each candidate variant, eVIDENCE performs filtering using the new BAM file. (**b**) Number of detected single nucleotide variants (SNVs) and insertions and deletions (indels) from cell-free DNA (cfDNA) sequencing data processed by Connor (left) and after applying eVIDENCE (right). The expected number of SNVs and indels are indicated by blue and red dotted line (70 and 5, respectively). (**c**) Number of detected variants among 6 cfDNA samples in which matched tumor sequencing data were available. An initial variant calling using the processed data by Connor detected 11806 variants containing 14 tumor variants (left). After applying eVIDENCE, a large number of candidate variants were discarded, but 12 tumor variants remained (right), showing that eVIDENCE efficiently filtered candidate variants.

We describe here the eVIDENCE method, designed to identify low-frequency variants and reduce false positive calls from sequencing data of the ThruPLEX tag-seq library. We developed two bioinformatics approaches in our method (Fig. 1a). First, we found that most candidate variants detected from the processed BAM file using Connor were located at either end of reads. As shown in Supplementary Fig. S1, unique molecular tags (UMTs) and stem sequences are ligated to both ends of DNA molecules. When a part of these artificial sequences is marked “alignment match”, instead of “soft-clipping” in the BAM CIGAR field, sequence mismatches can be introduced in the region. Therefore, we removed UMT and stem sequences and matched base qualities from raw BAM files and extracted UMT information (see “Methods” and Supplementary Methods). We kept the extracted UMT information by adding it to the read name. Using the new read names, new segment sequences and base qualities, we created new FASTQ files and mapped these to the reference genome sequence to generate new BAM files. Second, from the newly-produced BAM files, we extracted reads covering each position of the candidate variant and their UMT information, and grouped them into “UMT families”. A “UMT family” is a group of reads which have the same UMT, considered to originate from the same DNA molecule. If there were two or more reads that do not support the consensus base call within each UMT family, the candidate variant was discarded.

In order to validate the algorithm for this filtering, we generated an artificial library by mixing three libraries with different proportions (0.5% of RK442, 1.0% of RK443 and 98.5% of RK445). There were a total of 150 known single nucleotide polymorphisms (SNPs) that were present in either or both RK442 and RK443, but not in RK445. Theoretically, the VAF of the 150 SNPs in the mixed library was 0.25–1.5%. We analyzed the sequencing data of this library with eVIDENCE. Of the 150 positions, 144 were covered by variant-supporting raw reads, and the reads were grouped into UMT families. 105 positions had UMT families which contained two or more variant-supporting raw reads (see “Supplementary Methods”). We examined the base calls within each UMT family at these 105 positions, and detected UMT families which had two or more reads that do not support the consensus base call at seven positions. However, we succeeded in consensus base calling at 98 (93.3%) positions and our algorithm properly worked for making consensus reads.

### Application of eVIDENCE to cfDNA analysis from HCC patients

In the current study, 27 plasma samples were collected from 26 patients with HCC. A summary of patient characteristics is shown in Supplementary Table S2. Plasma was obtained at the time of recurrence or prior to liver resection, and cfDNA was extracted. Mean cfDNA concentration in plasma was 76.8 ng/mL. For each sample, 10 ng of cfDNA was used for library preparation. Each library was hybridized to our custom capture panel (Supplementary Table S1), and sequencing was performed at 6,800x average coverage (Supplementary Table S3). After removing duplicates, the average sequencing depth of each sample was 550x (Supplementary Table S3). We then identified candidate SNVs and short indels with VAF of ≥0.1% and with consensus reads to support the alteration of ≥3 using the processed BAM file. We detected a mean of 1,354 SNVs and 345 indels per sample (Supplementary Fig. S2). The average number of somatic point mutations and short indels were previously reported as 4.2 and 0.3 per megabase from whole-genome analysis of 27 HCCs^21^ and the targeted region of our custom panel was about 0.63 megabase. Therefore, the expected number of SNVs and indels were 2.6 and 0.19 per sample, respectively. This implies that the detected candidate variants retained a large number of false positives.

**Figure 2:**
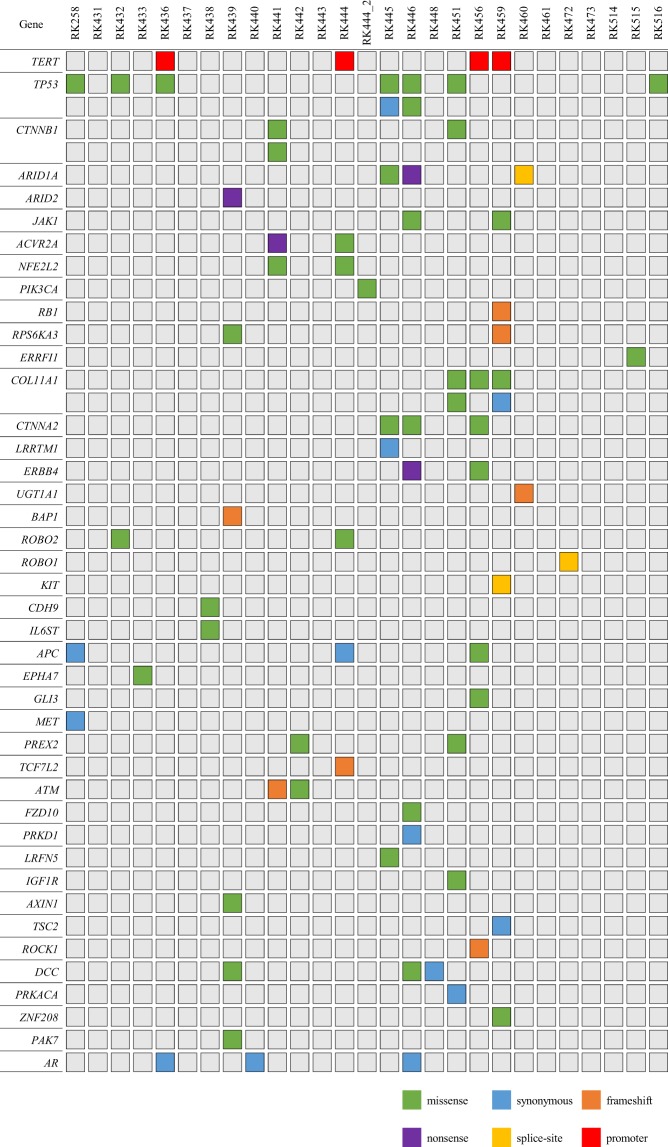
The landscape of genomic alterations in 27 cell-free DNA samples. Each column represented a sample and each row represents a gene. Color legends of the aberrations represent including missense, nonsense, synonymous, splice site, frameshift and promoter variant.

Application of eVIDENCE filter removed a large number of candidates, and finally, 50 nonsynonymous, 3 splice-site variants, 7 short coding indels and 13 synonymous variants were detected from the 27 samples. We also identified four *TERT* promoter hotspot (chr5: 1295228) variants (Fig. 2, Table 1 and Supplementary Table S4). The numbers of the remaining SNVs and indels were highly consistent with those expected (70 and 5, respectively) (Fig. 1b). Of the 26 tumor samples, whole-exome sequencing (WES) was performed on RK258 and targeted sequencing was conducted for RK436, RK441, RK442, RK444 and RK445. These tumor sequencing data revealed that an initial variant calling of the cfDNA samples contained 14 tumor variants. Of the 14 variants, 12 remained after applying eVIDENCE method (Fig. 1c). These findings showed that eVIDENCE could reduce the number of candidate variants efficiently.

**Table 1:** The variant allele frequency (VAF) distribution of the detected variants.

type	VAF (%)				total
	<0.5	0.5–1.0	1.0–5.0	5.0<	
nonsynonymous	22	15	8	5	50
synonymous	4	3	5	1	13
splice-site	0	2	0	1	3
indels	1	1	1	4	7
*TERT* promoter	0	1	3	0	4
total	27	22	17	11	77

Of the 77 variants identified across the 27 cfDNA samples, 49 (63.6%) showed VAF of <1% (0.20–0.98%) (Table 1). *TP53* was the most frequently altered gene and all the nine variants were located in the DNA binding domain that is encoded by exons 5 to 8 (Supplementary Table S4). Distribution of the VAF of driver genes of HCC^22–25^ are also shown in Table 2. About half of the driver gene variants were with VAF < 1%.

**Table 2:** The variant allele frequency (VAF) distribution of driver genes of hepatocellular carcinoma.

Gene	VAF (%)				total
	<0.5	0.5–1.0	1.0–5.0	5.0<	
*TERT*	0	1	3	0	4
*TP53*	2	4	1	2	9
*CTNNB1*	2	0	1	0	3
*ARID1A*	1	0	2	0	3
*ARID2*	0	0	1	0	1
*JAK1*	1	0	1	0	2
*ACVR2A*	0	0	1	1	2
*NFE2L2*	0	2	0	0	2
*PIK3CA*	1	0	0	0	1
*RB1*	0	0	0	1	1
*RPS6KA3*	0	0	1	1	2
total	7	7	11	5	30

### Validation of detected variants

To validate the identified variants, 25 SNV positions were selected in an unbiased manner and analyzed by targeted amplicon sequencing of matched tumor and lymphocyte samples (Table 3 and Supplementary Table S5). The validation revealed that four variants with VAF range of 0.21–0.67%, and eight with VAF of ≥1% were observed in tumor samples. However, 13 SNVs with VAF of <1% were not detected. To further validate these variants, we selected eight variants with VAF range of 0.25–0.80% and tested them by digital PCR of cfDNA and genomic DNA from matched tumor and lymphocyte. This analysis showed that all the tested variants were identified in cfDNA (Table 3 and Supplementary Fig. S3). Importantly, two of the eight variants were also detected in lymphocyte DNA (Supplementary Fig. S3), suggesting that these variants in cfDNA were not tumor-derived, but normal lymphocyte-derived. Nevertheless, we found no false positives, indicating that eVIDENCE had high specificity for detecting variants with 0.2% minimum allele fractions in cfDNA.

**Table 3:** Summary of 25 single nucleotide variants subjected to validation experiments.

Gene	Sample	Chr	Genomic position	Reference	Variant	AA change	Total number of consensus reads	Number of variant reads	VAF (%)	Validation with tumor DNA by amplicon sequencing	Validation with cfDNA by digital PCR
*TERT* promoter	RK436	5	1295228	G	A	—	395	6	1.52	y	—
*TP53*	RK432	17	7577120	C	T	R273H	884	7	0.79	n	N/A
*TP53*	RK451	17	7577133	T	C	S269G	1,179	3	0.25	n	y
*TP53*	RK258	17	7578503	C	T	V143M	1,216	106	8.72	y	—
*TP53*	RK436	17	7578535	T	G	K132T	620	61	9.84	y	—
*CTNNB1*	RK451	3	41274886	A	G	Q379R	1,376	22	1.60	y	—
*ARID1A*	RK445	1	27106316	C	T	S1976F	1,035	3	0.29	n	y
*ARID2*	RK439	12	46231342	T	G	Y394X	816	17	2.08	y	—
*ACVR2A*	RK441	2	148657079	G	T	E106X	696	8	1.15	y	—
*NFE2L2*	RK444	2	178098809	T	C	E79G	622	5	0.80	n	y
*NFE2L2*	RK441	2	178098956	A	C	L30R	751	5	0.67	y	—
*RPS6KA3*	RK439	X	20193353	T	A	S386C	397	10	2.52	y	—
*COL11A1*	RK456	1	103405977	G	A	P1097L	1,264	4	0.32	n	N/A
*COL11A1*	RK451	1	103488365	G	T	P393Q	1,384	10	0.72	n	N/A
*CTNNA2*	RK445	2	80097000	G	A	R175H	1,079	4	0.37	y	—
*ROBO2*	RK432	3	77571995	G	T	M292I	685	3	0.44	y	—
*CDH9*	RK438	5	26902700	G	T	P380T	1,453	3	0.21	y	—
*APC*	RK456	5	112175232	G	A	R1314K	1,174	5	0.43	n	y
*EPHA7*	RK433	6	93956601	C	T	E879K	615	4	0.65	n	N/A
*GLI3*	RK456	7	42004860	C	A	A1271S	1,263	7	0.55	n	N/A
*PREX2*	RK442	8	69104007	C	T	A1466V	857	27	3.15	y	—
*ATM*	RK442	11	108121480	T	G	C430G	677	5	0.74	n	y
*LRFN5*	RK445	14	42356455	G	C	K209N	996	4	0.40	n	y
*IGF1R*	RK451	15	99251289	A	G	N198S	1,275	8	0.63	n	y
*PAK7*	RK439	20	9561315	A	C	L156R	788	6	0.76	n	y

Note: AA, amino acid; VAF, variant allele frequency; cfDNA, cell-free DNA; y, successfully validated; n, NOT validated; N/A: not assessed due to a lack of sample volume for the experiment.

### Assessment of analytical sensitivity

**Figure 3:**
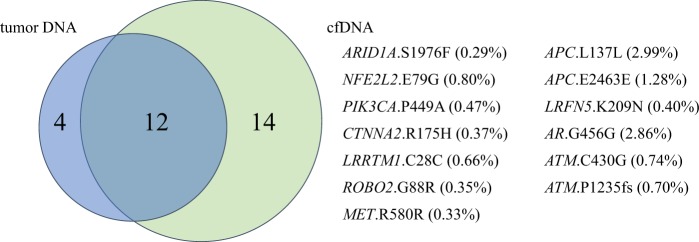
Concordance of genomic alterations in tissue and cell-free DNA (cfDNA) among 6 samples. Twelve out of 16 variants in tumor DNA were detected in cfDNA. Of 26 variants in cfDNA, 14 were detected in cfDNA, but one *TP53* variant was validated by the targeted amplicon sequencing of the tumor. Thirteen variants detected in cfDNA only and their variant allele frequency are shown. Driver gene variants such as *AIRD1A*.S1976F, *NEE2L2*.E79G and *PIK3CA*.P449A were also observed. *ATM*.C430G was detected in matched lymphocyte DNA by digital PCR.

We performed WES on one tumor sample and targeted sequencing on five samples and reviewed the tumor sequencing data to assess the sensitivity of our method. Among these six samples, we detected 16 variants in the genes of the tumor samples with our custom capture panel (Supplementary Table S6). On the other hand, a total of 26 variants were identified among the cfDNA samples (Fig. 3). Of the 16 variants identified in tumor samples, 12 (75%) were also detected in cfDNA, suggesting that our pipeline properly worked in identifying variants in tumor. In the 14 cfDNA specific variants, one *TP53* variant was subjected to validation by targeted amplicon sequencing of the tumor, and was successfully validated (see above). In the remaining 13 variants that were detected only in cfDNA, four variants were validated by digital PCR (Supplementary Fig. S3). Since the validation experiments by amplicon sequencing and digital PCR showed high specificity of our workflow, the rest of nine variants were likely to be true.

### Detection of HBV integration sites and rearrangements in the *TERT* region

We detected HBV integration sites and genomic rearrangements in the *TERT* region using read-pair information (See “Methods”). Across the 27 samples, seven HBV integration breakpoints and four structural variations in the *TERT* region were identified (Supplementary Tables S7 and S8). All the HBV integration was validated by breakpoint PCR, but only one *TERT* rearrangement was validated (Supplementary Fig. S4). Although validated translocation was supported by read pairs covering the rearrangement breakpoint, the others were supported by only two UMT families and their reads did not cover the breakpoints (Supplementary Table S8), which suggests that these calls might be false positives.

## Discussion

The analysis of ctDNA is an emerging strategy for noninvasive cancer diagnosis, monitoring of disease as well as molecular profiling. Although there are several reports that detect ctDNA with high sensitivity and specificity, effective analysis methods for identifying low-frequency variants of cfDNA have yet to be established. In the present study, we performed targeted capture sequencing of cfDNA from HCC patients using a custom gene panel and analyzed the data with eVIDENCE to detect rare variants. We used a commercially-available molecular barcoding kit (ThruPLEX Tag-seq) and the customized gene panel for creating sequencing libraries. The sequencing data were processed by an open source software specific for the kit (Connor), and candidate variants were detected from the processed data. Since the ThruPLEX tag-seq library has UMT and stem sequences on both 5′ and 3′ ends as shown in Supplementary Fig. S1, a large number of false positive calls were found in stem sequences instead of biological sequences. Each sequencing read contains the barcode and stem region on one end, and there are some reads which cover several bases of the stem region on the other side because cfDNA is highly fragmented. In the current study, 8.0% of the total number of consensus reads had stem sequences on the other side. Since it is very difficult to remove all exogenous sequence from such reads before alignment, we removed artificial sequences from the raw BAM file. We then examined whether the candidate variants existed in the newly-created BAM files. In addition, we performed further filtering by examining the base calls within each UMT family at the positions of the candidate variants because it has been reported that sequencing errors can be identified by comparing the reads containing the same barcode^26^. In our workflow, if there were two or more reads that do not support the variant call within each UMT family, the candidate variant was considered an error and discarded. The sequencing analysis of the mixed library revealed that consensus base calling was correctly done at 98 of 105 (93.3%) known variant positions, suggesting that the algorithm for this filtering was practical.

After implementing these modified bioinformatics approaches, we finally detected 70 SNVs and 7 indels across the 27 samples and 49 of them (63.6%) showed VAF of <1% (0.20–0.98%). The validation tests revealed the limit of variant detection of eVIDENCE was as low as 0.2%. In the sensitivity assessment of our method, we detected four tissue-specific variants among 16 detected in tumor DNA. We reviewed the sequencing data of the cfDNA samples for these four variants, and revealed that the depth of consensus reads was 90 for the *PTEN* variant, and the *TERT* promoter variant had one supporting UMT family (the cutoff value of the depth of consensus reads and the number of supporting UMT family was 100 and 3, respectively; see “Methods”). If there were more sequencing bases or more input cfDNA, these two variants would be identified in the cfDNA samples. The other two tumor specific variants were not listed as candidate variants due to low VAF in raw cfDNA sequencing data and therefore not found in cfDNA variants. In the analysis of the mixed library, we found most variant-supporting UMT families had one or two raw reads due to the very low proportions of RK442 and RK443, making it difficult to correctly perform variant calling (see “Supplementary Methods”). Although it was difficult to assess the sensitivity of our workflow with the artificial library sequencing, it revealed that consensus base calling was accurately done, suggesting that our pipeline properly worked in filtering candidate variants. These results demonstrate that eVIDENCE is highly accurate for detecting variants with 0.2% minimum allele fractions from comprehensive cfDNA sequencing data targeting about 80 genes using a custom panel. Our workflow provided filtering for errors caused by short fragment length of cfDNA and the structure of the ThruPLEX tag-seq library. Analysis of the filtered data with additional statistical methods considering base error type and sequence context may improve the sensitivity for low frequency variant detection. Furthermore, many other methods identifying rare variants^6–11,13,14^ interrogated a limited number of loci or targeted fixed genomic regions, while our pipeline can be applied to ctDNA analysis using barcoded sequencing libraries prepared with the ThruPLEX tag-seq kit and any custom gene panel targeting a large number of loci.

We identified 13 variants that existed in cfDNA, but not in tumor DNA from comparison analysis, and three of these were located in driver genes (*ARID1A*, *NFE2L2* and *PIK3CA*; Fig. 3). This indicates that ctDNA variants reflected a more comprehensive genomic profile of cancer patients, and that ctDNA examination using eVIDENCE could be more clinically useful than tissue analysis. However, lymphocyte variants can also be detected (*ATM* gene; Fig. 3). Importantly, in the current study, not all the detected variants in cfDNA were derived from tumors as two variants confirmed by digital PCR were also detected in matched normal lymphocyte samples (Supplementary Fig. S3). Mayrhofer *et al*.^27^ recently examined genomic profiles of cfDNA and matched lymphocyte DNA from 217 metastatic prostate cancer patients and showed that clonal hematopoiesis with somatic mutations caused false positive findings in cfDNA in 14.6% of patients. Therefore, careful assessment of variants is required when applying ctDNA analysis to clinical practice. If the variants detected are located in hotspots or annotated variants in reference databases such as the Catalog of Somatic Mutations in Cancer (COSMIC) (https://cancer.sanger.ac.uk/cosmic), they are likely to be tumor variants. If not, it can be helpful to examine whether identified variants are distributed in functionally important domains. For example, in the current study, all *TP53* variants were located in the DNA binding domain and known variant patterns in COSMIC, suggesting they are all tumor-derived variants. Additionally, it may be informative to estimate functional effects of non-hotspot variants by identifying mutation clusters in the protein tertiary structure as it has been reported that tumor mutations are enriched in the 3D protein structure among known driver genes^28,29^. On the other hand, understanding the mutation profile of white blood cells (WBC) is also important. Xia *et al*.^30^ examined the background somatic mutations in cfDNA from non-cancer individuals and reported the average mutant allele frequency of 50 cancer-associated genes in cfDNA. They showed the 7th most mutated gene was *ATM*, which is in line with our present finding. It may be possible to distinguish tumor mutations from somatic mutations of WBC according to the location of the variants if more data on background WBC mutations is made available.

In the present study, we also detected and validated seven HBV integration and one rearrangement in the *TERT* region from cfDNA analysis. It is reported that HBV integration into host genome is an early event which occurs prior to tumor development^31^, and that *TERT* translocations activate *TERT* expression, likely promoting carcinogenesis^24^. To our knowledge, no study has examined HBV integration or *TERT* rearrangements using comprehensive cfDNA sequencing data from HCC patients. Our results might be underestimating HBV integration breakpoints and structural variations since we performed targeted panel sequencing, not whole-genome sequencing. Nonetheless, considering the importance of HBV DNA integration and *TERT* rearrangements in HCC carcinogenesis, detecting these aberrations with cfDNA could be a useful analysis which leads to early diagnosis of HCC.

Despite several advantages in this study, there are some limitations. First, our workflow is specific to the ThruPLEX tag-seq library analysis, and it cannot be applicable to analysis of other types of barcoded libraries whose UMT is not tagged at the end of the read or stem sequence is not contained. Second, we could not experimentally validate 52 out of the 77 candidates due to a lack of sample volume. However, the VAF of the 52 candidates were not significantly different from those of the validated candidates (Supplementary Fig. S5). Therefore, we consider that the 52 candidates would also be detected with high accuracy. Finally, the average depth of consensus reads was relatively low (550x), which may not be sufficient to detect very low frequency variants. Despite the insufficient depth of coverage, our study successfully identified 12/16 variants found in tumor samples, suggesting that our method can work properly for analyzing cfDNA. However, greater depth of consensus reads is required to achieve higher sensitivity.

In conclusion, we demonstrated the clinical utility of ctDNA analysis using our approach in the HCC field. In addition, eVIDENCE can be applied to examine cfDNA from other types of malignancies using any custom gene panel, and could be helpful for developing precision medicine for HCC and other tumor types through liquid biopsies.

## Methods

### Ethics statement

This study was approved by the ethical committees at RIKEN, Hiroshima University and Wakayama Medical University (IRB approval numbers are 20–11, 26–13 and 66, respectively). All individuals have given written informed consent for research and publication. The experimental methods in this study were performed in accordance with the relevant guidelines and regulations.

### Clinical samples

Twenty-six patients with HCC were recruited at Hiroshima University and Wakayama Medical University during the period between 2014 and 2017. The patients’ clinical and pathological features are in Supplementary Table S2. From each patient, a blood volume of about 10 ml was collected in an EDTA-containing tube and plasma was obtained by two-step centrifugation (3,500 rpm for 10 minutes and 12,000 rpm for 10 minutes). The plasma was stored at −80 °C until cfDNA preparation. Plasma cfDNA was extracted from 1–2 ml of plasma using the QIAamp circulating nucleic acid kit (Qiagen) according to the manufacturer’s instructions. The concentration of the extracted cfDNA was measured by Qubit fluorometer (Thermo Fisher Scientific). Genomic DNA was also extracted from fresh-frozen tumor specimens and lymphocytes.

### Library preparation and targeted cfDNA sequencing

We prepared cfDNA sequencing libraries with unique molecular tags using ThruPLEX Tag-seq according to the manufacturer’s instructions. For each specimen, 10 ng of cfDNA was used for library preparation. We performed targeted sequencing using Agilent SureSelect XT Custom (Agilent Technologies) and an Illumina HiSeq. Our custom gene panel captures the exonic regions of 79 genes, *TERT* promoter region and chr18:56119000–56120500 (Supplementary Table S1). Then, 500–750 ng of purified library was hybridized to the capture panel for 16 or 24 hours at 65 °C. The subsequent library amplification and purification were performed according to the Agilent SureSelect XT Custom protocols. Purified products were examined by Bioanalyzer 2100 (Agilent Technologies) to evaluate their quality and quantity. Targeted sequencing was performed using paired-end 2 × 150 bp sequencing on HiSeq2500 (Illumina).

### Analysis of sequencing data

#### Candidate somatic variants calling

Sequencing reads were aligned against the human reference genome (GRCh37) using Burrows-Wheeler Aligner (BWA)^32^ and converted into BAM files by SAMtools^33^.

We processed the BAM files tagged by the ThruPLEX Tag-seq using Connor, an open source bioinformatics analysis tool. Connor de-duplicates a tagged BAM file and produces a new BAM file with consensus alignment pairs. The result files were converted into pileup format by SAMtools.

For candidate SNVs detection, the following criteria were applied; 1) VAF of ≥0.1% after removing base calls with base quality or mapping quality of <20; and 2) minimum number of variant-supporting consensus reads of 3. In addition, at each candidate SNV position, forward strand reference and variant alleles, as well as reverse strand reference and variant alleles were counted, respectively. Then, strand bias was calculated by two-sided Fisher’s exact test and candidate SNVs with the P-value of <0.001 were discarded. Candidate short indels were identified using the following criteria; (1) frequency of indels ≥0.1% after removing reads with mapping quality of <20; and (2) minimum supporting consensus reads of 3.

#### Production of new FASTQ files

The ThruPLEX Tag-seq Kit adds two 6 nucleotide UMTs and two 8–11 nucleotide non-random stems on each end of the cfDNA fragment (Supplementary Fig. S1). Therefore, each query sequence begins with the leading UMT and stem sequences, followed by the target sequence region and then, occasionally, the stem on the other end. BWA marks the UMT and stem sequence regions as “S (soft clipping)” and the target area as “M (alignment match)” in the CIGAR field of BAM files. However, when a part of stem sequence adjacent to the target is highly consistent with the reference genome, the region can be labeled as “M” with/without “I (insertion to the reference)” or “D (deletion from the reference)” operation. This behavior can introduce sequence mismatches in the stem regions whose origins are not biological molecules.

Therefore, we removed UMT and stem sequences and matched base qualities from the segment sequence and base quality fields of BAM files containing only reads covering the positions of the candidate variants. The UMT sequence was added to each read name for UMT information retention. New FASTQ files were produced using the new read names, sequences and base qualities. A detailed explanation is provided in the Supplementary Methods.

#### Filtering of candidate SNVs

The new FASTQ files were converted into BAM files and each position of the candidate SNV was examined using this new BAM file. We extracted reads covering the positions of the candidates from the newly-produced BAM files. Then, base calls at candidate positions with quality of ≥20 and the same UMT were grouped into a “UMT family”. UMT families with less than three base calls were discarded. In the default setting for Connor, for each family, a consensus sequence requires a 60% majority in the base call sequence at each position. This means if there are six same-variant calls and four reference calls at a position within a UMT family, the consensus sequence is determined as the variant. For more stringent criteria to reduce false positives, we discarded a candidate if there were two or more reads that did not support the variant call within each UMT family. Then, candidate SNVs with fewer than 100 UMT families or fewer than three support UMT families were excluded. After the filtering, candidates that were registered in dbSNP (http://www.ncbi.nlm.nih.gov/SNP/) or the integrative Japanese Genome Variation Database (http://ijgvd.megabank.tohoku.ac.jp/) were excluded. We also discarded candidates with VAF of >20%. Finally, the remaining SNVs were functionally annotated with ANNOVAR^34^.

#### Filtering of candidate indels

We filtered indels using the new BAM files in a similar approach used for the SNV filtering, and reads covering the candidate positions were selected. We then extracted the CIGAR values, as well as MD:Z tags, which describe mismatching positions and sequences and UMTs. Reads with the same UMT were grouped together and UMT families with fewer than three reads were discarded. Then, if there were two or more CIGAR values which did not support the majority CIGAR within each family, the candidate indel was discarded. The MD:Z tag was used to confirm if the UMT family supported the candidate indel or not. Finally, indels with 100 or more UMT families and more than two supporting UMT families were included, and functionally annotated with ANNOVAR.

### Validation of the algorithm for consensus base calling

To validate the algorithm for filtering candidate variants described above, we generated an artificial library by mixing three libraries with different proportions (0.5% of RK442, 1.0% of RK443 and 98.5% of RK445). We performed sequencing with a depth of consensus reads of 1,000x, and analyzed the data using eVIDENCE. A detailed explanation is provided in the Supplementary Methods.

### Validation of variants by targeted amplicon sequencing

To validate the identified variants, tumor DNA and their corresponding lymphocyte DNA were amplified for the selected 25 SNVs using the primers shown in Supplementary Table S5, and amplicon libraries were prepared. Sequencing was performed on MiSeq (Illumina). The average read depth at the candidate positions was 351,400x and 325,000x for the tumor and lymphocyte samples, respectively. We measured the difference in the allele frequencies of the variants between the tumor and matched normal samples by one-sided Fisher’s exact test and the cutoff P-value for significance was determined as 0.001 (Supplementary Table S5).

### Digital PCR analysis for validation

For eight candidate SNVs that were not detected by targeted amplicon sequencing of the tumor DNA, the fractional abundance of variant alleles in cfDNA and genomic DNA from matched tumor and lymphocyte was analyzed by the QuantStudio 3D Digital PCR system (Thermo Fisher Scientific) according to the manufacturer’s protocols. The primers and probes are listed in Supplementary Table S9.

### Whole-exome sequencing and targeted capture sequencing of tumor DNA

To compare variants of cfDNA with those of tumor DNA, we performed WES on one tumor sample and targeted sequencing on five samples. DNA was extracted from frozen tumor tissues and lymphocytes, and 1 μg of DNA was sheared to 200 bp peak target size. After adapter ligation and amplification, the purified library was hybridized to the Agilent SureSelect Human All Exon v6 chip (Agilent Technologies) or Agilent SureSelect XT Custom. Sequencing was performed on HiSeq2500 (Illumina) and mutation analysis was conducted using Genomon2 (https://genomon-project.github.io/GenomonPagesR/).

### HBV integration and *TERT* rearrangement calls

To detect HBV integration, sequencing reads were mapped to the human (GRCh37) and HBV reference genome (GenBank accession: NC_003977.1). We discarded read pairs in which both reads were perfectly aligned to human or HBV genome and selected paired-end reads in which one read was mapped to the human genome and the other to the HBV genome. Read pairs that had the same UMT were grouped into UMT family and candidate integration sites supported by two or more families were used for validation. We performed breakpoint PCR validation of these candidates, and all of them were successfully validated.

To identify *TERT* rearrangements, we selected read pairs in which one read was mapped to *TERT* and its promoter region (chr5: 1253846–1305107) and the other was aligned to another chromosome or location with a distance from the paired read of >1 kb. We grouped alignment pairs that share the same UMT and UMT families within 300 bp were clustered. Clusters supported by two or more UMT families were determined as candidate *TERT* rearrangements. PCR validation test was performed and only one of four candidates was validated.

## Supplementary information

Supplementary Materials

Dataset 1

## Data Availability

The source code of eVIDENCE is freely available from https://github.com/mizunokei/eVIDENCE (10.5281/zenodo.2567667). The sequencing data is available upon request.
