# Supplementary material for: eVIDENCE: a practical variant filtering for low-frequency variants detection in cell-free DNA
Source: Sci Rep. 2019 Oct 22;9:15017. doi: 10.1038/s41598-019-51459-4 (PMC6805874; doi:10.1038/s41598-019-51459-4)
Supplement: Supplementary file 1 — Supplementary Materials [file 41598_2019_51459_MOESM1_ESM.pdf]

Supplementary Materials

eVIDENCE: a practical variant filtering for low-frequency variants detection in cell-free DNA

Kei Mizuno, Shusuke Akamatsu, Takayuki Sumiyoshi, Jing Hao Wong, Masashi Fujita, Kazuaki Maejima, Kaoru Nakano, Atushi Ono, Hiroshi Aikata, Masaki Ueno, Shinya Hayami, Hiroki Yamaue, Kazuaki Chayama, Takahiro Inoue, Osamu Ogawa, Hidewaki Nakagawa* and Akihiro Fujimoto*

Correspondence should be addressed to Akihiro Fujimoto (afujimoto@m.u-tokyo.ac.jp) and Hidewaki Nakagawa (hidewaki@ims.u-tokyo.ac.jp).

Supplementary Methods

Supplementary Figures: Figure S1–S6

Supplementary Methods

Generating new FASTQ files

The ThruPLEX Tag-seq Kit adds two 6 nucleotide unique molecular tags (UMTs) and two 8–11 nucleotide non-random stems on each end of the cfDNA fragment (Supplementary Fig. S1). Therefore, each query sequence begins with the leading UMT and stem sequences, followed by the target sequence region and then, sometimes, the stem on the other end. Burrows-Wheeler Aligner (BWA)[1] marks the UMT and stem sequence regions as “S (soft clipping)” and the target area as “M (alignment match)” in the CIGAR field of BAM files. However, when a part of stem sequence adjacent to the target is highly consistent with the reference genome, the region can be labeled as “M” with/ without “I (insertion to the reference)” or “D (deletion from the reference)” operation. This behavior can introduce sequence mismatches in the stem regions whose origins are not biological molecules.

We removed UMT and stem sequences and matched base qualities from the segment sequence and base quality fields of BAM files containing only reads covering the positions of the candidate variants as described below.

1) When a read is a forward read, the first 6 bases in the query sequence field represent UMT. UMT sequence was kept as left UMT for later use.
2) The following stem sequence is determined uniquely by the beginning base. For example, when it starts with “A”, the sequence is “AGTAGCTCA” (Supplementary Fig. S1). If the hamming distance between the expected stem sequence and the query sequence was ≥ 2, the read was discarded.
3) When a stem sequence is “AGTAGCTCA”, the CIGAR value should start with “15S”, due to the first 6 bases and the following 9 bases being from UMT and the stem, respectively. However, there were several inconsistent values which we divided into the following cases:
    A) The CIGAR values start with “xS” and $x < 15$

    When the first CIGAR operation was S and its length was less than 15, the length was

changed to 15 and 15-*x* was subtracted from the length of the following operation. We also added 15-*x* to the leftmost mapping position.

B) The CIGAR values start with "*x*S" and $x > 15$

When the first CIGAR operation was S and its length was more than 15, the length was changed to 15 and *x*-15 was added to the length of the following operation. We also subtracted *x*-15 to the leftmost mapping position. However, if there were extra "GTAGCTCA", a common sequence of four kinds of stems, in the following sequence, the region of S operation was extended to the position of the rightmost common sequence. The length of the following operation and the mapping position was changed accordingly. This duplication of stem sequences can occur during library synthesis.

C) The CIGAR values start with "*x*M" and $x < 15$

Firstly, we added the lengths of M and I operations sequentially until the sum gave 15 or more. Here we defined sum of the lengths of M and I operations as sum_MI, and sum of the lengths of M and D operations as sum_MD.

When sum_MI was 15, there were two cases in which the next operation was D or M. For example, in the former case, the CIGAR value could start with "*x*M*y*D*z*I*w*D" (sum_MI = $x+z$ = 15, sum_MD = $x+y$). We changed this value to "15S" and added sum_MD+*w* to the mapping position. In the latter case, the CIGAR value could be "*x*M*y*D*z*I*w*M" (sum_MI = $x+z$ = 15, sum_MD = $x+y$). We changed this value to "15S*w*M" and added sum_MD to the mapping position.

When sum_MI was more than 15, there were also two cases in which the last CIGAR operation when sum_MI exceeded 15 was M or I. For example, in the former case, the CIGAR value could be "*x*M*y*D*z*I*w*M" (sum_MI = $x+z+w > 15$, sum_MD = $x+y+w$). We changed this value to "15S(sum_MI-15)M" and added (15+sum_MD-sum_MI) to the mapping position. In the latter case, the CIGAR value could be started with "*x*M*y*D*z*I" (sum_MI = $x+z > 15$, sum_MD = $x+y$). We changed this value to "15S(sum_MI-15)I"

and added sum_MD to the mapping position.

D) The CIGAR values start with "*x*M" and $x = 15$

When the first CIGAR operation was M and its length was 15, the following operation must be D or I. Namely, the CIGAR value starts with "15M*y*D" or "15MyI". In the former case, we changed the M operation to S and deleted D operation. We then added 15+*y* to the leftmost mapping position. In the latter case, we changed the M operation to S and added 15 to the mapping position.

E) The CIGAR values start with "*x*M" and $x > 15$

When the first CIGAR operation was M and its length was more than 15, the value was changed to "15S(*x*-15)M". We then added 15 to the leftmost mapping position.

F) A) ~ E) was performed when a stem sequence started with "T", "G" and "C" (the length of S operation should be 17, 14 and 16, respectively).

4) When a read is a reverse read, the last 6 bases in the query sequence field represent UMT and the adjacent 8–11 bases indicate a stem sequence. The same procedure mentioned above was done for reverse reads, but the leftmost mapping positions were not changed. UMT sequence was also kept as right UMT.

5) Bases and base qualities in the newly modified soft clipping regions were removed from the segment sequence and base quality fields of BAM files.

6) The leftmost and rightmost mapping positions were compared between paired reads. The rightmost mapping position was calculated by adding (sum_MD-1) to the leftmost mapping position (sum_MD: sum of the lengths of M and D operations in the revised CIGAR value). If the rightmost position of a forward read was larger than that of a reverse read, the forward read was considered to cover the stem sequence on the 3' side. Similarly, if the leftmost position of a reverse read was smaller than that of a forward read, the reverse read was considered to cover the stem sequence on the 5' side. In these cases, bases originated from stems and matched base qualities were removed.

Finally, for paired reads, left and right UMT sequences extracted from forward and reverse reads were added to the read name. Using the newly created read names, segment sequences and base qualities, a new FASTQ file was produced. This file was converted into BAM format by BWA and SAMtools[2] for further filtering of candidate variants.

Filtering of candidate variants

For variants filtering, reads covering the position of each candidate variant with mapping quality ≥ 20 were selected. If three or more variants were found, or the distance between two variants was less than 10 bp in any reads, the reads were all discarded. We then extracted base calls with quality ≥ 20 and UMTs at each candidate position for single nucleotide variant (SNV) filtering. Base calls which share the same UMT were grouped into a "UMT family". In the same way, CIGAR values, MD:Z tags and UMTs were extracted for indel filtering, and CIGAR and MD:Z were coordinated into UMT families. UMT families in which the number of family members of ≤ 2 were discarded. When the number of UMT families, which represents the coverage of each candidate position, was less than 100, the candidate variant was discarded.

Next, the consensus base call or CIGAR value was determined by majority within each family. If there were two or more family members which did not support the consensus in any UMT families, we discarded the candidate. For indel filtering, the MD:Z tag was used for confirming whether the UMT family supported the candidate indel or not.

Furthermore, we checked the UMTs of families which supported candidate variants. If left or right UMT matched exactly among families supporting a variant, the candidate was discarded. In addition, if UMT of one family was consistent with combination of left UMT and right UMT of other two families, the family was discarded as this family might be generated by the recombination of the other two families. Finally, if the number of families supporting a variant was less than three, the candidate variant was discarded.

For further filtering of candidate SNVs, candidates represented in dbSNP

(http://www.ncbi.nlm.nih.gov/SNP/) or the integrative Japanese Genome Variation Database (http://ijgvd.megabank.tohoku.ac.jp/) were excluded. Additionally, we examined the distribution of variant allele frequency (VAF) of candidate SNVs registered in public databases with those of other candidates (Supplementary Fig. S6). This revealed that the latter had a bimodal distribution whereby one peak existed in a region of < 11%, and the other existed in a region of > 22%, which was mostly consistent with one peak of the former one near 40%. This suggested that there was clustering of heterozygous single nucleotide polymorphisms. For this reason, we regarded SNVs with VAF of > 20% as germline variants and discarded them. After these filtering, the remaining variants were functionally annotated with ANNOVAR[3].

Validation of the algorithm for consensus base calling

To validate the algorithm for filtering candidate variants described above, we generated an artificial library by mixing three libraries with different proportions (0.5% of RK442, 1.0% of RK443 and 98.5% of RK445). There were a total of 150 known single nucleotide polymorphisms (SNPs) that were present in either or both RK442 and RK443, but not in RK445. This mixed library was sequenced and analyzed with eVIDENCE. Of the 150 positions, 144 were covered by variant-supporting raw reads and the reads were grouped into UMT families. However, most variant-supporting UMT families had one or two raw reads due to the very low proportions of RK442 and RK443. Since each variant-supporting UMT family in RK442 and RK443 libraries had only 10–20 raw reads, the number of variant-supporting reads with the same UMT became extremely low when the libraries were diluted. The number of raw reads in each variant-supporting UMT family was only one at 39 positions and one or two at 72 positions. Only 33 positions had UMT families which contained three or more variant-supporting raw reads. We examined the base calls within each UMT family at the105 positions that had UMT families which contained two or more variant-supporting raw reads to check whether consensus base calling was correctly done.

References

1. Li, H. & Durbin, R. Fast and accurate short read alignment with Burrows-Wheeler transform. *Bioinformatics* **25,** 1754–1760 (2009).
2. Li, H. *et al.* The Sequence Alignment/Map format and SAMtools. *Bioinformatics* **25,** 2078–2079 (2009).
3. Wang, K., Li, M. & Hakonarson, H. ANNOVAR : functional annotation of genetic variants from high-throughput sequencing data. *Nucleic Acids Res.* **38,** e164 (2010).

Supplementary Figures

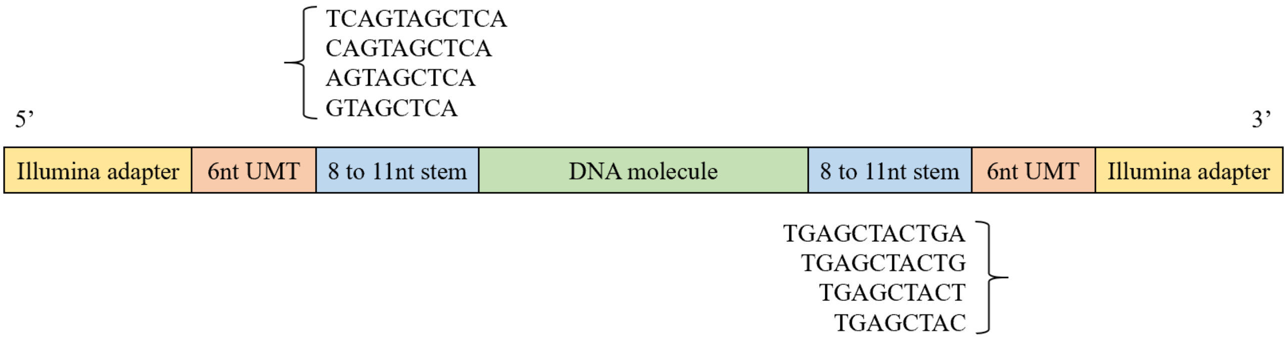

Figure S1: Structure of the DNA library using the ThruPLEX Tag-seq. Two 6-nucleotide unique molecular tags (UMTs) and two 8–11 nucleotide stems are added on each end of the DNA molecule and the Illumina adapters are then ligated. The stem sequences are shown.

a

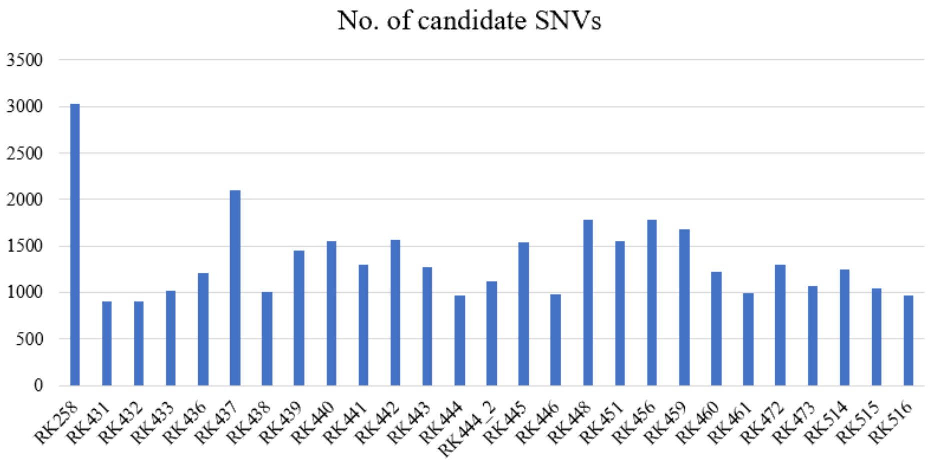

b

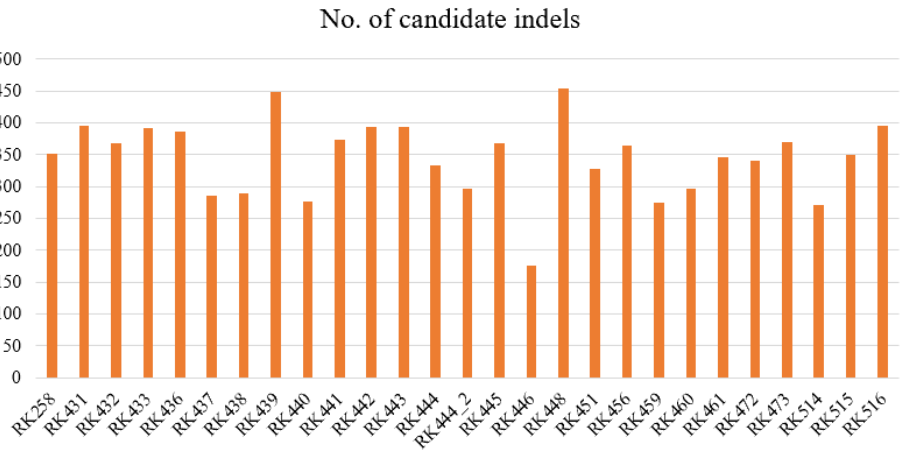

Figure S2: The number of the initial candidate single nucleotide variants (SNVs) (a) and short insertions and deletions (indels) (b) across 27 samples. A mean of 1354 candidate SNVs and 345 short indels were called.

a *TP53* S269G

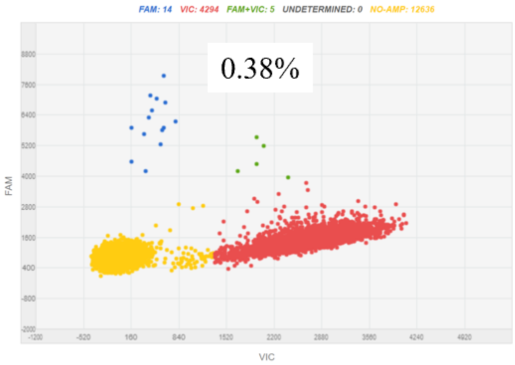

cfDNA

b *ARID1A* S1976F

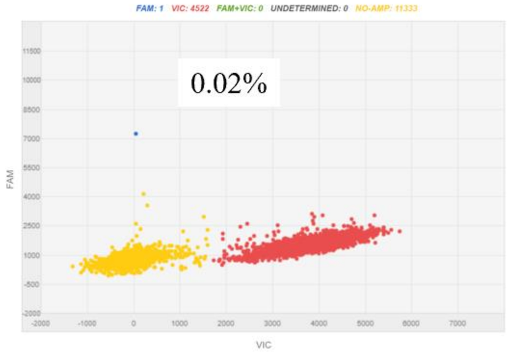

cfDNA

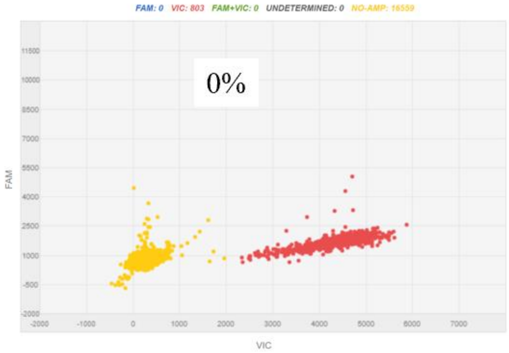

tumor DNA

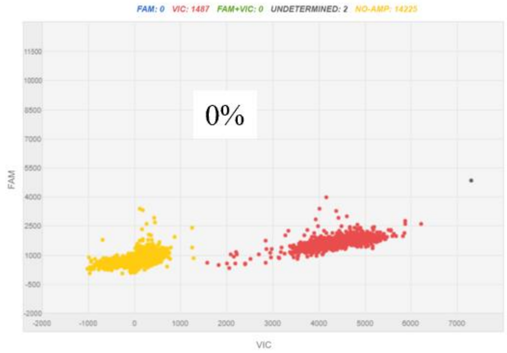

lymphocyte DNA

c *NFE2L2* E79G

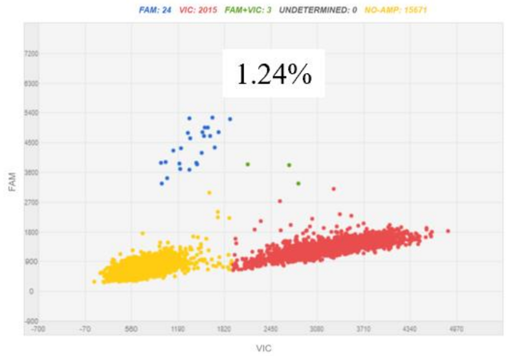

cfDNA

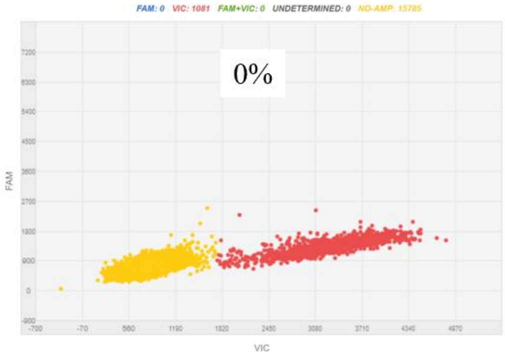

tumor DNA

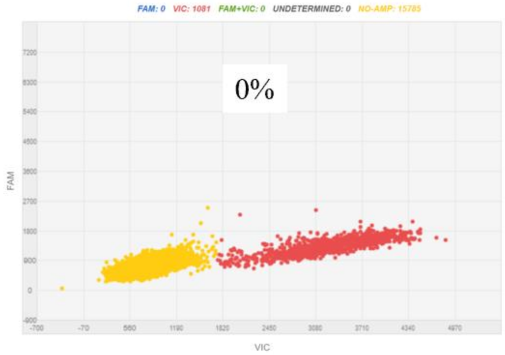

lymphocyte DNA

d *APC* R1314K

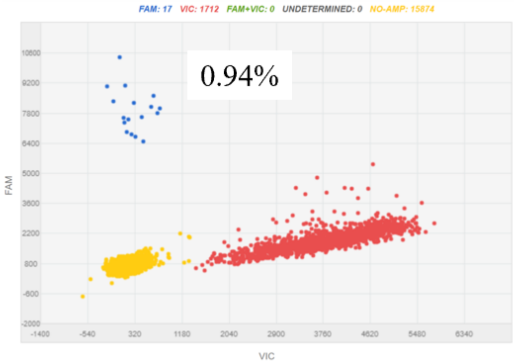

cfDNA

e *ATM* C430G

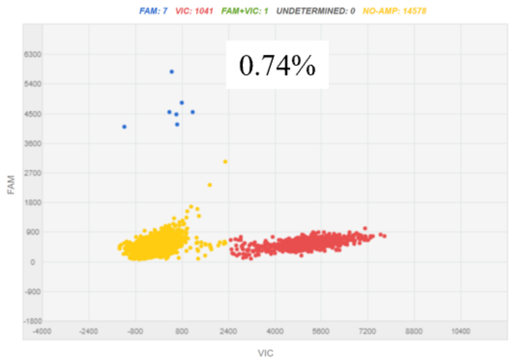

cfDNA

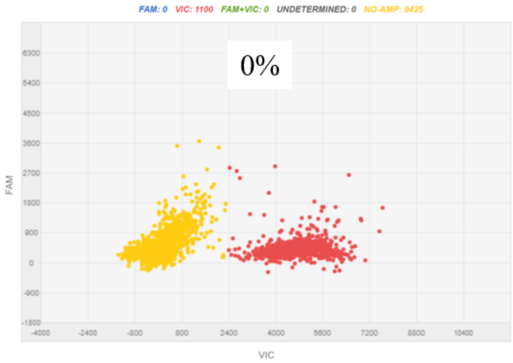

tumor DNA

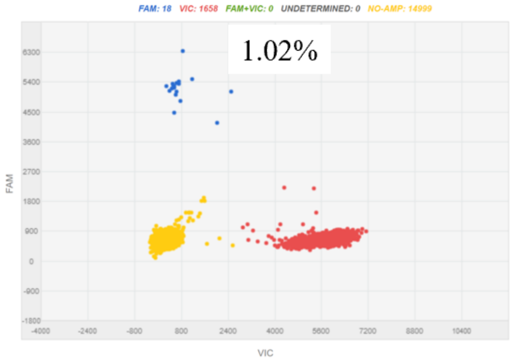

lymphocyte DNA

f *LRFN5* K209N

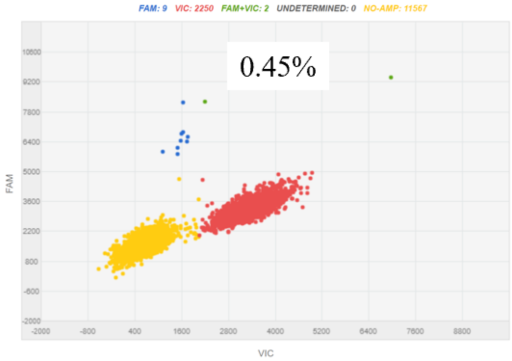

cfDNA

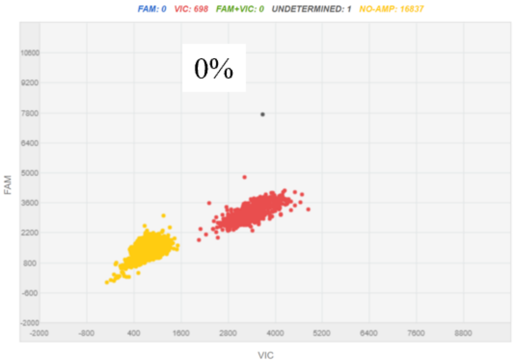

tumor DNA

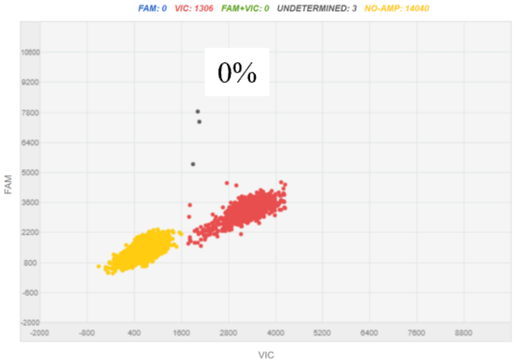

lymphocyte DNA

g *IGF1R* N198S

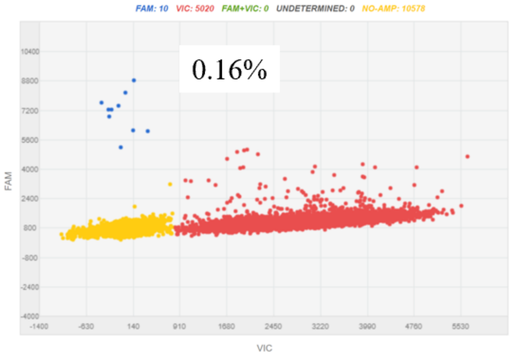

cfDNA

h *PAK7* L156R

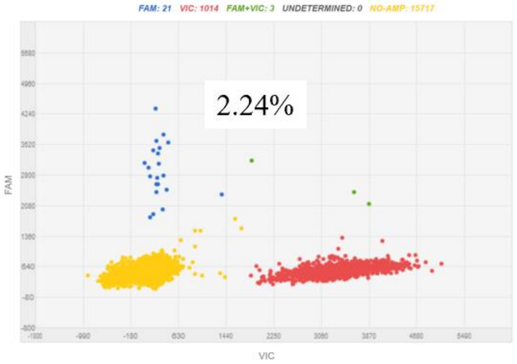

cfDNA

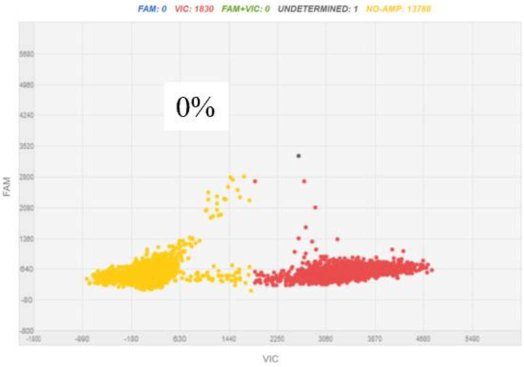

tumor DNA

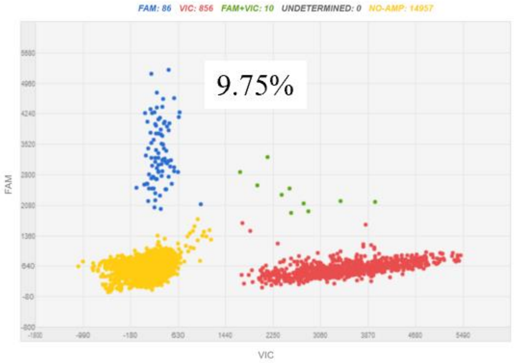

lymphocyte DNA

Figure S3: The results of validation by digital PCR of the cell-free DNA (cfDNA) samples. For each image, blue points indicate high FAM fluorescence intensity and show variant alleles. Red points indicate high VIC fluorescence intensity and show wild-type alleles. Green dots show the mix of FAM and VIC channel positive wells. Yellow points show empty wells without fluorescence. Variant allele frequency is also shown. The tumor DNA and lymphocyte DNA samples were not available for the assay of *TP53*.S269G (a), *APC*.R1314K (d) and *IGF1R*.N198S (g). Two variants of *ATM*.C430G (e) and *PAK7*.L156R (h) were found to be lymphocyte variants. *ARID1A*.S1976F (b), *NFE2L2*.E79G (c) and *LRFN5*.K209N (f) were detected only in cfDNA and reflected tumor heterogeneity.

a HBV integration

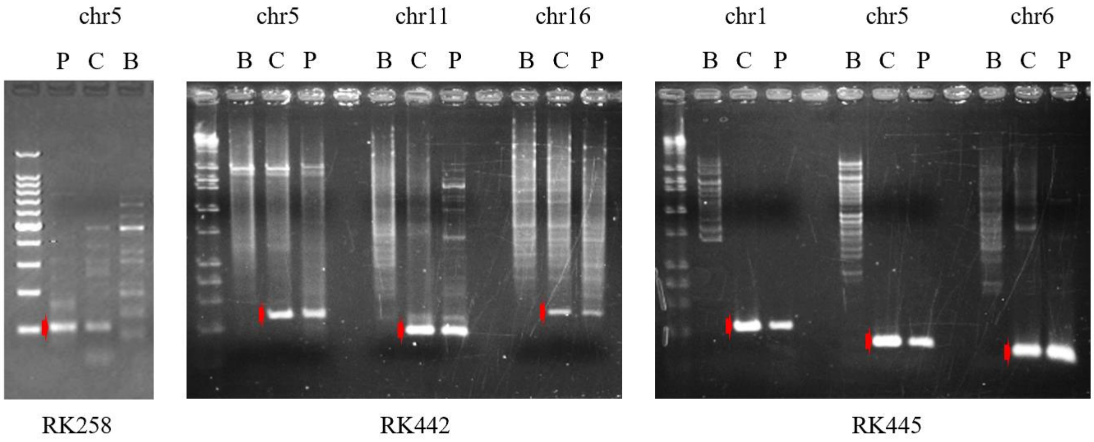

b *TERT* rearrangement

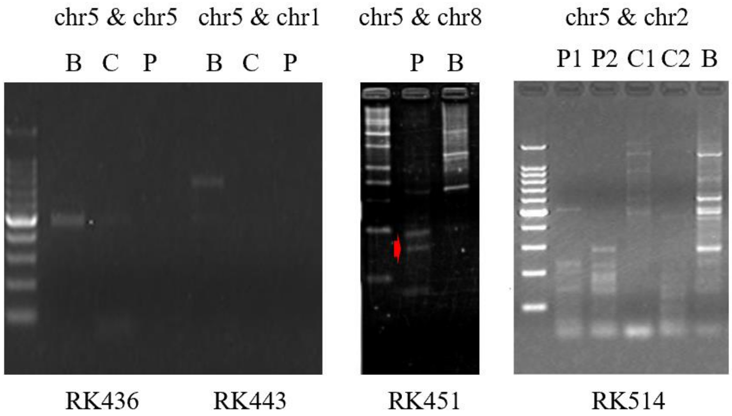

Figure S4: Gel electrophoresis of breakpoint PCR for detecting HBV integration (a) and *TERT* rearrangements (b). Cell-free DNA (cfDNA) (P) and DNA extracted from lymphocyte samples (B) and tumors (C) were amplified with the use of primers designed to detect breakpoints. The red arrows show the target products. (a) All the seven HBV integration sites across the three samples were detected in cfDNA and tumor DNA, but not in lymphocyte DNA. (b) Only one out of four *TERT* rearrangements identified by data analysis was detected in cfDNA of RK451. The tumor DNA of RK451 was not available. Different gels were prepared as separate figures.

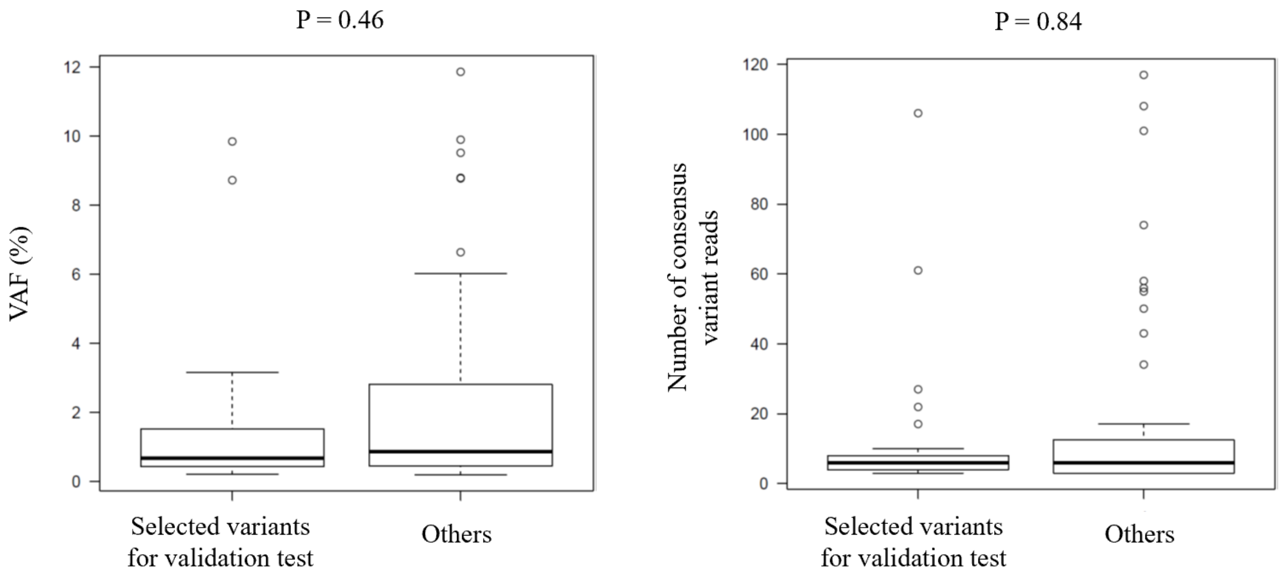

Figure S5: Box plots comparing the distribution of the variant allele frequency (VAF) (left) and the number of consensus variant-supporting reads (right) between 25 variants selected for the validation experiments and the other 52 variants. There was no significant difference between the two groups, suggesting that the validation results of the selected variants would be similar to those of the others.

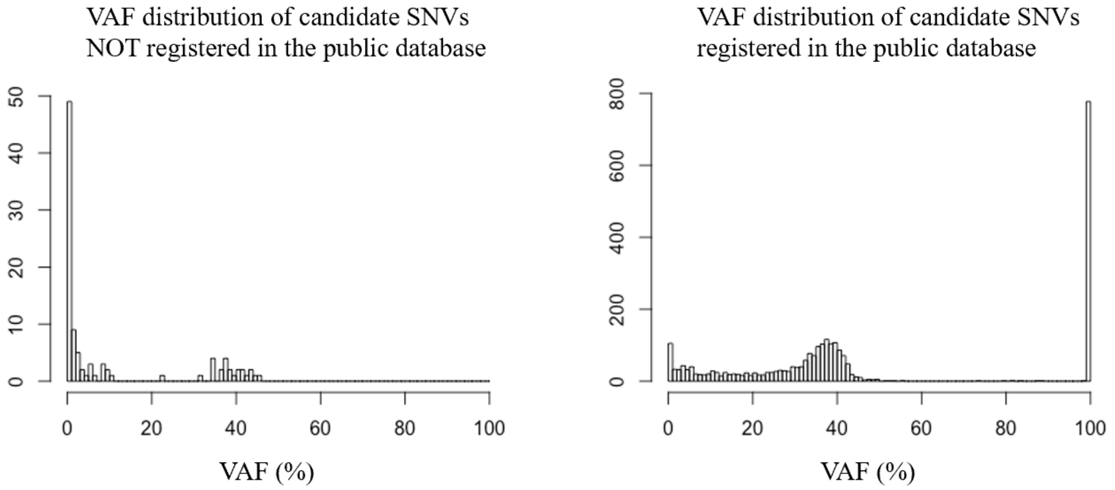

Figure S6: The variant allele frequency (VAF) distribution of candidate single nucleotide variants (SNVs) before final filtering. The VAF distribution of candidate SNVs that were unregistered in public databases (left) had a bimodal distribution whereby one peak existed in a region of < 11% and the other existed in a region of > 22%. The VAF distribution of candidates that are registered in public databases (right) had two peaks around 40% and at 100%, indicating heterozygous and homozygous single nucleotide polymorphisms (SNPs), respectively.
